# Asbestos: a hidden player behind the cholangiocarcinoma increase? Findings from a case–control analysis

**DOI:** 10.1007/s10552-013-0167-3

**Published:** 2013-02-14

**Authors:** Giovanni Brandi, Stefania Di Girolamo, Andrea Farioli, Francesco de Rosa, Stefania Curti, Antonio Daniele Pinna, Giorgio Ercolani, Francesco Saverio Violante, Guido Biasco, Stefano Mattioli

**Affiliations:** 1 Department of Experimental, Diagnostic and Specialty Medicine, Sant’Orsola-Malpighi Hospital, University of Bologna, Via Massarenti, 9, 40138 Bologna, Italy; 2 Department of Medical and Surgical Sciences, University of Bologna, Bologna, Italy; 3 Department of Liver and Multi-organ Transplantation, Sant’Orsola-Malpighi Hospital, University of Bologna, Bologna, Italy

**Keywords:** Asbestos, Cholangiocarcinoma, Occupational exposure, Occupational diseases, Case–control studies, Bile duct neoplasms

## Abstract

**Purposes:**

We conducted a case–control analysis to explore the association between occupational exposure to asbestos and cholangiocarcinoma (CC).

**Methods:**

The study was based on historical data from 155 consecutive patients with CC [69 intrahepatic CC (ICC) and 86 extrahepatic CC (ECC)] referred to Sant’Orsola-Malpighi University Hospital between 2006 and 2010. The cases were individually matched by calendar period of birth, sex, and region of residence to historical hospital and population controls. Occupational exposure to asbestos was retrospectively assessed considering job titles obtained from work histories. Separate conditional logistic regression models were applied for ECC and ICC. Estimates were adjusted for smoking status and socioeconomic class.

**Results:**

We matched 149 controls (median birth year: 1947; males: 56 %) to 41 cases of ICC (median birth year: 1946; males: 56 %) and 212 controls (median birth year: 1945; males: 48 %) to 59 cases of ECC (median birth year: 1945; males 51 %); 53 cases were not matched due to residence or birth year. We found an increased risk of ICC in workers exposed to asbestos (adjusted OR 4.81, 95 % CI 1.73–13.33); we also observed suggestive evidence that asbestos exposure might be associated with ECC (adjusted OR 2.09, 95 % CI 0.83–5.27). Sensitivity analysis restricted to patients from the Province of Bologna produced confirmatory figures.

**Conclusions:**

Our findings suggest that ICC could be associated with asbestos exposure; a chronic inflammatory pathway is hypothesized. Exposure to asbestos could be one of the determinants of the progressive rise in the incidence of ICC during the last 30 years.

## Introduction

Cholangiocarcinoma (CC), a rare malignancy arising from cholangiocytes (the epithelial cells lining the biliary tree), is the second most common primary liver malignancy, accounting for up to 25 % of primary liver tumors [1]. Anatomically, the CCs are commonly divided into intrahepatic (ICC) and extrahepatic (ECC) forms each presenting different epidemiological features.

A progressive increase in the incidence and mortality of CC, namely ICC, was reported worldwide (with the exception of Denmark) in the last quarter of twentieth century. The current ICC incidence has now reached or even overtaken that of ECC which instead has remained stable or slightly decreased [2–4]. This trend has also been observed in Italy where ICC mortality considerably increased (from 0.01 to 0.59/100,000) from 1980 to 2003, overtaking the incidence of ECC [5].

The ICC increase recorded in recent decades seems to be a true phenomenon rather than the effect of improved diagnostic techniques, since it is not associated with significant changes in early stage cancer diagnosis [6]. In addition, the increasing incidence of ICC was confirmed after taking into account the possible misclassification of hilar cholangiocarcinomas (Klatskin tumors), a form of ECC cross-referenced to ICC in the second edition of the International Classification of Diseases for Oncology [7]. Further, the ICC increase does not seem to have reached a plateau and regard all age groups.

The broad geographic variations in incidence probably reflect a different distribution of local risk factors, suggesting that putative carcinogenetic factors could have a spatial–temporal segregation. In particular, the increased incidence of ICC in developed countries began after the 1980s and was mainly observed in males and in elderly patients [8–10].

Several case–control studies designed to clarify these epidemiological features investigated the risk factors linked to CC [1, 11, 12]. The findings disclosed that some risk factors are involved in both ICC and ECC development, whereas others are more specific to one of the two forms. Bile duct diseases (primary sclerosing cholangitis, primary biliary cirrhosis, choledochal cysts, choledocholithiasis, cholecystitis, and liver flukes), primarily affecting large intrahepatic bile ducts and/or extrahepatic bile ducts, contribute to both CC forms though there are large differences in odds ratios. Inflammatory bowel diseases, alone or via primary sclerosing cholangitis, also serve to accrue both ICC and ECC incidence.

Cholelithiasias and prior cholecystectomy are recognized risk factors mainly for ECC, whereas hepatolithiasis, obesity, and chronic liver disease (hemochromatosis, non-alcoholic steatohepatitis, and hepatitis C virus or hepatitis B virus infection with or without cirrhosis) are only involved in ICC [1, 13].

In developed countries, most cases of CC occur in the absence of known risk factors [1]. Therefore, other undefined possibly environmental and/or occupational factors could be involved in the remaining two-thirds of cases and are probably responsible for the recent ICC increase.

Asbestos exposure has sometimes been implicated in the development of CC [14, 15]. The biological rationale of asbestos carcinogenesis in the biliary system is based on the following factors:

1. asbestos fibers can be drained by convective flow into initial pulmonary lymphatics; once they reach the blood through the lymphatic system, asbestos fibers can potentially translocate to all organs dragged by water fluxes down pressure gradients [16];
2. major fiber deposition has been found in the liver due to the high microvascular permeability of the liver sinusoids [15, 17];
3. asbestos fibers in the liver can give rise to a chronic inflammatory status with production of oxygen radicals, cytokines, and growth factors leading to impaired cell proliferation and apoptosis [18].

Our case–control analysis aimed at exploring the association between occupational asbestos exposure and risk of CC development.

## Methods

### Selection of cases

This study was based on a cohort of 155 consecutive patients with histologically confirmed CC (69 with ICC and 86 with ECC) referred to Sant’Orsola-Malpighi University Hospital (Bologna, Italy) between January 2006 and December 2010. The hospital represents a referral center for the treatment of liver malignancies. The catchment area of the hospital extends beyond the regional limits of Emilia-Romagna, and many patients are referred from other parts of Italy.

### Selection of controls

We used a historical comparison group consisting of controls sampled in three other case–control studies: 211 subjects were enrolled among population controls sampled from the Italian health service registries to study carpal tunnel syndrome [19]; 62 among hospital controls of a study on renal cell carcinoma previously conducted at Sant’Orsola-Malpighi University Hospital [20]; eight among hospital controls of a study on rhegmatogenous retinal detachment conducted among ophthalmic outpatients of Bologna [21].

Our target was to match each individual case to four controls based on calendar period of birth (5-year interval), sex, and region of residence (Italy is administratively divided into 20 Regions). Unfortunately, population controls were available only for nine Italian Regions: Emilia-Romagna, Lombardy, Marche, Apulia, Sardinia, Tuscany, Trentino Alto Adige, Umbria, and Veneto. Therefore, cases from other Italian Regions were not matched and were excluded from our analysis.

Since 54 out of 155 cases (34.8 %) were from the Province of Bologna, a special rule for matching was applied to these cases. Specifically, they were matched to controls of the same Province instead of the same Region (the Emilia-Romagna Region comprises eight other provinces in addition to Bologna).

In each matching stratum, we randomly drew up to four controls for each case. Since we studied ICC and ECC separately, controls were sampled independently for the two pathologies; thus, one control could have been matched to both an ICC and an ECC case. Due to availability of controls, we were not always able to reach the one to four ratio between cases and controls.

### Exposure assessment and classification of confounders

For controls, data on birth year, sex, region of residence, smoking status, and complete occupational history were obtained from the already filled in structured questionnaires.

Information for cases available from clinical records included the following: birth year, sex, region of residence, smoking status (never/ever), and life-prevalent (i.e., longest held) job title. In 2011, we telephonically contacted the CC cases to take a more detailed occupational history. Data were collected using a brief questionnaire derived from that used by Mattioli and colleagues [19]. The term asbestos was not mentioned in the questionnaire. Information for 71 (46 %) deceased subjects was collected from their relatives. We were unable to collect new information in seven (4.5 %) cases, so only clinical records were used for these subjects. For controls, data on birth year, sex, region of residence, smoking status, and complete occupational history were obtained from the already filled in structured questionnaires.

Occupational exposure to asbestos was assessed considering the entire job histories and calendar periods. Taking into account the time-dependent variation of the diffusion of asbestos use, exposed subjects were classified as those who had held at least one potentially exposed job during their working life. Assessment was performed independently by two raters (S.M. and A.F.) unaware of case/control status. In case of disagreement between the two raters, the subject was classified as occupationally exposed to asbestos. Interrater agreement was very good (kappa statistics 0.94).

Because of the small number of exposed subjects in our population (*n* = 54), exposure to asbestos was classified as a dichotomous variable. Moreover, since information on the occupational history of cases was limited to job titles and calendar period, an estimation of cumulative asbestos dose would have been unreliable.

We identified the life-prevalent (i.e., longest held) job title for each subject. This datum was used to assign the socioeconomic status that was classified according to the three classes of The National Statistics Socio-economic Classification derived by the simplified method [22]. The three broad socioeconomic classes were (1) managerial and professional occupations; (2) intermediate occupations (i.e., higher grade white collar workers, petit bourgeoisie or independents, and higher grade blue collar workers); (3) routine and manual occupations, and never worked and long-term unemployed. Smoking status was classified as a dichotomous variable (ever/never smokers).

### Statistical analysis

Analyses were performed using data obtained from CC cases and their matched controls. When data were missing, we used listwise deletion, excluding from the analysis four cases of ICC and three cases of ECC (with missing information on smoking status and/or occupation). We explored the association between CC and occupational exposure to asbestos performing separate analyses for ICC and ECC. We conducted a sensitivity analysis restricted to cases and controls from the Province of Bologna. Within this subpopulation, referral bias, if existing, is likely to be less pronounced than in the entire study population. Odds ratios (ORs) and relative 95 % confidence intervals (95 % CIs) were estimated using prospective logistic regression models conditioned on matching variables (birth year, sex, and region of residence) according to Breslow and Day [23]. All analyses were performed using Stata 11.2 SE (Stata corporation, Texas, TX, USA).

## Results

Two cases of ICC were excluded from the analysis since no information was available on their occupational history. Matching was not possible for 53 cases due to region of residence (21 ICC and 19 ECC) and birth year (5 ICC and 8 ECC).

After these exclusions, we matched 149 controls (median birth year: 1947; males: 56 %) to 41 cases of ICC (median birth year: 1946; males: 56 %) and 212 controls (median birth year: 1945; males: 48 %) to 59 cases of ECC (median birth year: 1945; males 51 %). The characteristics of the CC cases and of their matched controls are summarized in Table 1. No major differences were noted among cases and controls for socioeconomic status and smoking history. For occupational exposure to asbestos, we observed a different distribution among ICC cases and their matched controls. The distribution of known risk factors among CC cases is reported in Table 2. Most of our patients did not present any known risk factor for CC. Among ICC cases, the risk factor with the highest prevalence was infection with HBV or HCV. Among ECC patients, a history of hepato/cholelithiasis was recorded in 10 (17 %) patients.

**Table 1:** Intrahepatic and extrahepatic cholangiocarcinoma: characteristics of matched cases and controls

Characteristics	Intrahepatic cholangiocarcinoma				Extrahepatic cholangiocarcinoma			
	Cases		Controls		Cases		Controls	
	*N* [*n* = 41]	(%)	*N* [*n* = 149]	(%)	*N* [*n* = 59]	(%)	*N* [*n* = 212]	(%)
Sex								
Males	23	(56.1)	84	(56.4)	30	(50.8)	102	(48.1)
Females	18	(43.9)	65	(43.6)	29	(49.2)	110	(51.9)
Birth year								
1920–1929	2	(4.9)	8	(5.4)	2	(3.4)	8	(3.8)
1930–1939	9	(22.0)	36	(24.2)	18	(30.5)	60	(28.3)
1940–1949	14	(34.1)	46	(30.9)	18	(30.5)	69	(32.5)
1950–1959	9	(22.0)	34	(22.8)	13	(22.0)	46	(21.7)
1960–1969	5	(12.2)	20	(13.4)	4	(6.8)	15	(7.1)
1970–1979	2	(4.9)	5	(3.4)	4	(6.8)	14	(6.6)
Residence								
Bologna Province	20	(48.8)	80	(53.7)	32	(54.2)	121	(57.1)
Other parts of Italy	21	(51.2)	69	(46.3)	27	(45.8)	91	(42.9)
Smoking status								
Never smoker	20	(48.8)	78	(52.4)	34	(57.6)	111	(52.4)
Ever smoker	21	(51.2)	71	(47.6)	25	(42.4)	101	(47.6)
Socioeconomic status								
Managerial and professional occupations	12	(29.3)	51	(34.2)	23	(39.0)	76	(35.9)
Intermediate occupations	10	(24.4)	34	(22.8)	16	(27.1)	45	(21.2)
Routine and manual occupations	19	(46.3)	64	(43.0)	20	(33.9)	91	(42.9)
Occupational exposure to asbestos								
Not exposed	28	(68.3)	132	(88.6)	48	(81.4)	191	(90.1)
Exposed	13	(31.7)	17	(11.4)	11	(18.6)	21	(9.9)

**Table 2:** Distribution of known risk factors among patients with intrahepatic and extrahepatic cholangiocarcinoma

Risk factor	Intrahepatic cholangiocarcinoma		Extrahepatic cholangiocarcinoma	
	*N*	(%)^a^	*N*	(%)^a^
Viral hepatitis (HBC or HCV)	8	(20)	5	(8)
Cirrhosis	4	(10)	0	(0)
Alcoholic liver disease	2	(5)	1	(2)
Hepato/cholelithiasis	4	(10)	10	(17)
Primary sclerosing cholangitis	2	(5)	2	(3)
Congenital liver malformations	0	(0)	0	(0)
Liver flukes	0	(0)	0	(0)
Exposure to thorotrast	0	(0)	0	(0)
None of the above	22	(44)	41	(69)

*HBV* hepatitis B virus, *HCV* hepatitis C virus

^a^Percentages do not add up to 100 % since two or more risk factors may coexist

In our study population, 54 subjects were classified as previously exposed to asbestos. Professions associated with asbestos exposure included the following: airline mechanics (*n* = 1); auto mechanics/brake specialists (*n* = 5); blacksmiths/goldsmiths (*n* = 2); boiler workers (*n* = 1); carpenters (*n* = 3); construction workers (*n* = 11); furnace men (*n* = 1); insulators (*n* = 2); launders/ironers (*n* = 3); linotype technicians (*n* = 1); merchant mariners (*n* = 3); metal workers (*n* = 1); plumbers (*n* = 7); railroad workers (*n* = 4); road machine operators (*n* = 7); shipyard workers (*n* = 2). In fifty-two subjects, the potential exposure to asbestos occurred during the 1960s, 1970s, or 1980s—a period during which the asbestos pro-capita consumption in Italy was over 1,000 tons per million inhabitants [24]. Only two controls were first exposed in the early 1990s, just before the national asbestos ban in 1992.

Table 3 estimates the association between ICC and exposure (i.e., asbestos, smoking history, and socio-occupational status) derived from conditional logistic regression models. Occupational exposure to asbestos appeared to be strongly associated with ICC (at multivariate analysis, OR 4.81, 95 % CI 1.73–13.33), whereas no signs of association were found between ICC and socioeconomic status or smoking history. The estimates for ECC provided suggestive evidence of the association between the disease and occupational exposure to asbestos, even though only weak statistical evidence supports this finding (at multivariate analysis, OR 2.09, 95 % CI 0.83–5.27). Like ICC, ECC showed no clear sign of association with socioeconomic status or smoking history (Table 3).

**Table 3:** Odds ratios and 95 % confidence intervals of intrahepatic and extrahepatic cholangiocarcinoma

Exposure	Cases [*n* = 41]	Controls [*n* = 149]	Univariate analysis		Multivariate analysis	
			Odds ratio^a^	(95 % CI^a^)	Odds ratio^a^	(95 % CI^a^)
Intrahepatic cholangiocarcinoma						
Occupational exposure to asbestos						
Not exposed	28	132	1.00	Ref.	1.00	Ref.
Exposed	13	17	4.16	(1.67–10.39)	4.81	(1.73–13.33)
Smoking status						
Never smoker	20	78	1.00	Ref.	1.00	Ref.
Ever smoker	21	71	1.15	(0.58–2.30)	0.92	(0.44–1.92)
Socioeconomic status						
Managerial and professional occupations	12	51	1.00	Ref.	1.00	Ref.
Intermediate occupations	10	34	1.30	(0.52–3.25)	0.71	(0.25–2.06)
Routine and manual occupations	19	64	1.31	(0.55–3.08)	0.91	(0.36–2.25)

Exposure	Cases [*n* = 59]	Controls [*n* = 212]	Univariate analysis		Multivariate analysis	
			Odds ratio^a^	(95 % CI^a^)	Odds ratio^a^	(95 % CI^a^)
Extrahepatic cholangiocarcinoma						
Occupational exposure to asbestos						
Not exposed	48	191	1.00	Ref.	1.00	Ref.
Exposed	11	21	1.90	(0.79–4.60)	2.09	(0.83–5.27)
Smoking status						
Never smoker	34	111	1.00	Ref.	1.00	Ref.
Ever smoker	25	101	0.75	(0.40–1.42)	0.78	(0.40–1.50)
Socioeconomic status						
Managerial and professional occupations	23	76	1.00	Ref.	1.00	Ref.
Intermediate occupations	16	45	1.10	(0.51–2.37)	0.94	(0.43–2.08)
Routine and manual occupations	20	91	0.67	(0.33–1.36)	0.63	(0.30–1.31)

*95* *%* *CI* 95 % confidence intervals, *Ref* reference category

^a^Estimates from logistic regression models conditioned on matching variables (birth year, sex, and region of residence)

Table 4 shows the results of the sensitivity analysis conducted using only cases from the Province of Bologna along with their matched controls. Due to the small number of cases, only univariate analysis was conducted. Estimates of the association between ICC and ECC and occupational exposure to asbestos were in line with those obtained using the entire study population, whereas a decreased risk of ECC among subjects from the lowest socioeconomic class was present (OR 0.35, 95 % CI 0.12–0.99) when considering only subjects from the Province of Bologna.

**Table 4:** Odds ratios and 95 % confidence intervals of intrahepatic and extrahepatic cholangiocarcinoma

Exposure	Intrahepatic cholangiocarcinoma				Extrahepatic cholangiocarcinoma			
	Cases [*n* = 20]	Controls [*n* = 80]	Univariate analysis		Cases [*n* = 32]	Controls [*n* = 121]	Univariate analysis	
			Odds ratio^a^	(95 % CI^a^)			Odds ratio^a^	(95 % CI^a^)
Occupational exposure to asbestos								
Not exposed	14	71	1.00	Ref.	28	110	1.00	Ref.
Exposed	6	9	3.41	(1.01–11.54)	4	11	1.37	(0.40–4.67)
Smoking status								
Never smoker	8	43	1.00	Ref.	17	56	1.00	Ref.
Ever smoker	12	37	1.81	(0.64–5.09)	15	65	0.69	(0.29–1.65)
Socioeconomic status								
Managerial and professional occupations	5	25	1.00	Ref.	14	36	1.00	Ref.
Intermediate occupations	5	16	1.63	(0.38–6.92)	9	27	0.81	(0.29–2.27)
Routine and manual occupations	10	39	1.36	(0.37–5.08)	9	58	0.35	(0.12–0.99)

Analysis restricted to cases and controls from the Province of Bologna

*95* *%* *CI* 95 % confidence intervals, *Ref* reference category

^a^Estimates from logistic regression models conditioned on matching variables (birth year and sex)

## Discussion

In the present study, we found an increased risk of ICC in workers exposed to asbestos, irrespective of socioeconomic status, and smoking history. In addition, suggestive evidence of increased risk for ECC was found among workers exposed to asbestos. These findings suggest a putative role of asbestos in ICC pathogenesis and possibly in its increasing incidence.

In Italy, asbestos was used extensively for more than 100 years before it was banned in 1992. Because of the long lag time between exposure and disease development, we are now witnessing asbestos-related diseases such as mesothelioma and lung cancer [25].

Asbestos has been suspected as a risk factor for CC but never specifically investigated before the present study. In 1983, a case of bile duct cancer in a patient with asbestosis was described. At postmortem examination, after digesting tumor tissue, short asbestos bodies similar to those observed in the lung were recovered in the liver [15].

Among others, the Swedish Cancer-Environment Register reported an increased standardized incidence ratio (SIR) of CC in some asbestos-related occupations: wholesale building materials (SIR for men 2.3); shipbuilding and repair (SIR for women 7.3); insulation workers (SIR for men 10.6) [14]. The sample size of the study was able to disclose these associations as the Swedish Cancer-Environment Register collected data on all employed Swedish citizens, followed-up for 19 years (1961–1979). Conversely, most studies on asbestos exposure and cancer were performed on worker cohorts too small to disclose an increased incidence of such a rare tumor.

The presence of asbestos fibers in the bile ducts can be explained by their translocation pathway: the fibers can cross the alveolar barrier after inhalation or penetrate the gastrointestinal mucosa after ingestion. The fibers then reach the interstitial environment and circulatory system through lymphatic vessels and are finally delivered to all tissues, where they may start an inflammatory (and hence possibly malignant transformation) process [16]. The prerequisite for ICC pathogenesis is the presence of asbestos in the biliary tract, mainly in canals of Hering, bile ductules, and interlobular bile ducts that are the principal targets of carcinogenic agents. After their translocation from the circulatory system, asbestos fibers may remain trapped in the smaller bile ducts. This would explain why asbestos exposure seems to be involved only in ICC pathogenesis and probably not in ECC development, also considering that the multipotent stem cells putatively involved in carcinogenesis differ for ICC and ECC [11]. Once the asbestos fibers reach the bile ducts, they could give rise to ICC through a chronic inflammation pathway, the same mechanism activated by established risk factors. Inflammatory conditions promote carcinogenesis by producing reactive oxygen and nitrogen species from inflammatory and epithelial cells, activating reparative tissue proliferation and creating a local environment rich in cytokines and other growth factors, ultimately resulting in DNA damage [26].

### Limits

The present exploratory case–control analysis was initially based on a consecutive series of CC cases seen in our center and on historical controls. Due to its composition, the study population could be prone to referral bias. Thus, the different proportion of subjects occupationally exposed to asbestos among the ICC cases, and the population controls could be an artifact due to case selection. To address this possibility, we conducted a sensitivity analysis using only cases and controls from the Province of Bologna, a subset of our population in which a referral bias is less likely. Estimates obtained in the sensitivity analysis were in line with those obtained in the main analysis.

Because of the individual matching, we excluded from our analysis 53 cases of CC, mainly due to region of residence; hence our analysis was based on a small number of cases (41 ICC and 59 ECC). We decided to use an individual matching based on relatively small geographic areas (i.e., Italian Regions) to take into account the extreme variability and heterogeneity characterizing the economic structures in Italy (a different distribution of production activities is appreciable even among small adjacent areas). A matching based on a broader classification (e.g., North, Center, and South of Italy) would have prevented us excluding so many cases from the analysis. Nevertheless, we believe this kind of approach would have increased the risk of spurious findings, since the prevalence of subjects with a former occupational exposure to asbestos could be strongly influenced by geographical origin.

Another limitation of the present study is the retrospective assessment of occupational exposure to asbestos: we cannot exclude exposure misclassification. Unfortunately, we only had information on life-prevalent job titles for seven patients. In addition, since occupational histories were collected from relatives for 46 % of cases, it is likely that for these subjects, we missed some job titles held during their youth. On balance, occupational exposure to asbestos once in a lifetime could have been underestimated for cases compared to controls, for whom self-reported occupational histories were always available. However, were this the case, the association between occupational exposure to asbestos and CC would have been underestimated. The control group, mainly composed of population controls (75 %) and ophthalmic outpatients from the Province of Bologna (3 %) [19, 21], also included inpatients referred to Sant’Orsola-Malpighi University Hospital (22 %) [20]. Among these subjects, exposure to asbestos could have been slightly overestimated; hence, our estimates could have been biased toward the null hypothesis (Berkson’s bias). A possible limitation of our study arises from the time when information was collected. Cases and controls were matched by age but, due to the study design, data on controls were collected 6–10 years before data on cases. Therefore, an underestimation of the number of controls occupationally exposed to asbestos could be possible, due to the truncation of occupational histories. However, the mean age of the cases at the time of the interview was 56 years (SD 11 years), and all data were collected after the national asbestos ban, introduced in 1992. Hence, a first exposure to asbestos is unlikely to have occurred among previously unexposed controls between the time of the interview and 2011. On balance, we believe that underestimation of exposure among controls, although possible, should not have substantially biased our estimates. Since exposure assessment was conducted blindly in relation to case/control status, other sources of differential misclassification (information bias) appear unlikely. Under these conditions, the risk of CC is more likely to have been underestimated than overestimated (bias toward the null hypothesis). Data of controls were mostly collected by self-administered questionnaires, although telephone administration of the questionnaire was tried among non-responders [19, 20]. Information for cases was always collected by telephone interviews. The etiological hypothesis was not mentioned during the interview, and job titles—which can be considered non-sensitive data—were collected using a structured questionnaire. Under these conditions, the quality of information on occupational history is unlikely to have been strongly influenced by the mode of data collection.

Due to the study design (case–control analysis performed with already collected data), information on possible confounders was poor. For the controls, we had no information on established/putative risk factors of CC such as parasitic infections, primary sclerosing cholangitis, bile duct cysts, hepatolithiasis, toxins, chemical carcinogens, inflammatory bowel disease, hepatitis C virus, hepatitis B virus, cirrhosis, diabetes, obesity, alcohol intake, and host genetic polymorphisms [1, 2]. Our analysis took into account possible confounding by cigarette smoking, roughly classified as never/ever smoker. We also made at least partial adjustment for socioeconomic status, classified according to the three classes version of The National Statistics Socio-economic Classification. Application of the more informative classification in five or eight classes was not feasible due to the small study population [22].

## Conclusions

Our findings support the hypothesis that ICC could be associated with asbestos exposure, possibly through a chronic inflammatory pathway. We also observed suggestive evidence that asbestos exposure might be associated with ECC.

To confirm the possible role of asbestos exposure in ICC development, we are planning a larger population-based case–control study to collect information on asbestos exposure (occupational and non-occupational) and other known/putative ICC and ECC determinants.
